# Chemical, Physical, and Degradation Characteristics of Ryegrass Cultivars Grown in Autumn and Winter for Dairy Cows

**DOI:** 10.3390/ani13203158

**Published:** 2023-10-10

**Authors:** Xuezhao Sun, Ao Chen, Jianping Li

**Affiliations:** 1 The Innovation Centre of Ruminant Precision Nutrition and Smart Farming, College of Animal Science and Technology, Jilin Agricultural Science and Technology University, Jilin 132109, China; xuezhaos@hotmail.com; 2 Jilin Inter-Regional Cooperation Centre for the Scientific and Technological Innovation of Ruminant Precision Nutrition and Smart and Ecological Farming, Jilin 132109, China; 3 AgResearch Limited, Grasslands Research Centre, Private Bag 11008, Palmerston North 4442, New Zealand; 4 Food Experience and Sensory Testing (Feast) Laboratory, Massey University, Palmerston North 4442, New Zealand; a.chen2@massey.ac.nz

**Keywords:** autumn-saved pasture, composition, in situ degradation, ryegrass cultivars

## Abstract

**Simple Summary:**

New Zealand has a temperate climate, and dairy farming relies on ryegrass pasture-based systems, normally with a winter-calving system. Although there is adequate pasture growth in some regions during winter, dairy farmers use autumn-saved pasture to sustain cows for one to two months before and after calving. During the transition period at calving, dairy cows have major changes in both physiology and nutritional requirements. The knowledge of ryegrass cultivars would enable the most appropriate selection to benefit cows pre- and post-calving. However, studies on Winter ryegrass are scarce. In this study, we measured nine ryegrass cultivars, including perennial, hybrid, and Italian types grown in late autumn and winter and harvested after seven or nine weeks of regrowth. The crude protein concentration was similar across types, but perennial ryegrasses contained the most fibre. Italian ryegrasses had the greatest proportion of soluble nutrients. The degradation rate of insoluble (fibrous) nutrients was the highest in Italian and the lowest in perennial grasses. Ryegrasses grown during winter had lower crude protein and fibre and higher non-fibre carbohydrate concentrations than that grown in late autumn. In conclusion, this study′s insights into the characteristics of nine ryegrass cultivars, grown in different seasons, offer valuable guidance for selecting the most suitable options to support the nutritional requirements of dairy cows during the transition period around calving.

**Abstract:**

During winter and early spring, pasture supply is usually lower than the demand in New Zealand dairy farming systems and thus the ‘autumn saved pastures’ (stockpiling) are introduced to fill the gap. This study aimed to investigate the chemical, physical, and degradation characteristics of ryegrass pastures, the predominant forage in New Zealand, grown in autumn and winter. To serve as ‘autumn saved pasture’, nine ryegrass cultivars, comprising three types (three perennial, three hybrid, and three Italian), were grown in late autumn (Autumn) and early winter (Winter) and harvested after 7 and 9 weeks of regrowth, respectively. The experiment had two experimental factors: ryegrass type (or cultivar) and harvest season. These experimental factors were in a randomised block design with the forage plot as the experimental unit. The degradation characteristics were assessed in the rumen of fistulated cows using the in situ incubation technique. Perennial ryegrass had a greater neutral detergent fibre (NDF) concentration (468 g/kg dry matter (DM)) than the hybrid (435 g/kg DM) or Italian (414 g/kg DM) ryegrasses. Italian ryegrasses had the greatest soluble fraction of DM (64.2% vs. 46.7% and 40.7%) and the greatest degradation rate of an insoluble but degradable fraction of DM (0.221 vs. 0.189 and 0.145/h) than the hybrid and perennial ryegrasses. Compared with the Winter ryegrass, the Autumn ryegrass had a greater crude protein concentration (246 vs. 208 g/kg DM) and a greater NDF concentration (486 vs. 392 g/kg DM) but a lower calculated soluble carbohydrate concentration (152 vs. 263 g/kg DM). It is concluded that there are notable variations among the cultivars, highlighting distinctions in parameters, such as NDF concentration, soluble fractions, degradation rates, and nutrient content among the perennial, hybrid, and Italian ryegrasses, as well as the seasonal variations observed between autumn and winter growth. These findings will not only facilitate enhanced nutrition for dairy cows as they undergo the transition phase but also have practical implications for future research and dairy cow nutrition.

## 1. Introduction

Ryegrass is the predominant forage in New Zealand, accounting for over 80% of feed dry matter consumed by dairy cows [1]. Ryegrass pasture composition exerts a profound influence on voluntary dry matter (DM) intake, nutrients available for absorption, and ultimately livestock production [2,3,4,5]. Changes in the quality of ryegrass/clover swards, as affected by climatic conditions, have been summarised [6,7], and Litherland and Lambert [8] have documented the fluctuations in pasture composition in New Zealand. The concentrations of crude protein (CP) and neutral detergent fibre (NDF) exhibit significant variability [9], but the adoption of intensive pastoral management practices, such as rotational grazing, serves to circumvent the accumulation of dead matter in the sward, consequently minimising the fluctuations in chemical composition [10,11] and thereby maximising ruminant productivity [12].

Across New Zealand dairy farms, a substantial portion exceeding 60% of on-farm costs can be attributed to activities, like regrassing, supplementary feeding, and fertiliser application [13]. An understanding of pasture regrowth, quality, digestion, and absorption is imperative to achieving maximal production output and enhancing overall profitability. Perennial ryegrass chemical composition and in situ degradation (ruminal digestion) characteristics have been studied from 14 to 105 days of regrowth [14]. A similar experiment was conducted with three contrasting ryegrass cultivars, demonstrating significant effects of both the cultivar and regrowth stage on herbage chemical composition and in situ degradation [15]. Furthermore, Chaves, Waghorn, Brookes, and Woodfield [14] documented the limited effects of the initial cutting date on herbage chemical composition and degradation characteristics during early spring. During winter and early spring, pasture supply is usually lower than demand, and supplements, such as silage and dry feeds, are needed until the pasture growth is sufficient to meet cow requirements in the spring. Alternatively, the practice of ‘autumn saved pastures’ (stockpiling) is introduced to fill the gap, wherein the pasture is conserved for feeding dairy cows for 1 to 2 months pre- and post-calving during winter and early spring [16]. In addition, the slow pasture growth in cooler conditions leads to limited senescence accumulation [17]. Ryegrass types, including perennial (Perennial, *Lolium perenne* L.), hybrid (Hybrid, *L. perenne × Lolium multiflorum* L.), and Italian (Italian, *L. multiflorum*) ryegrasses, have demonstrated adaptability, resilience, forage quality, and growth over other species in cold environments [18,19,20]. A better understanding of chemical composition and degradation characteristics will guarantee that appropriate supplements are chosen for dairy cows during winter.

The timing of the winter harvest as a dominating factor affects the formation of dry matter yield and the digestibility of organic matter, along with nutrient concentration in the herbage. Notably, Goliński et al. [21] delineated a strategy whereby the management of winter grazing systems could ensure that the crude protein and energy concentration of tested autumn-saved herbage align with the requirements of suckler cows or beef cattle until the culmination of the year if pre-utilised in July. In light of these considerations, the objective of this study was to evaluate diverse ryegrass cultivars grown in autumn and winter for the chemical composition and degradation characteristics of these ryegrasses in autumn-saved pastures.

## 2. Materials and Methods

### 2.1. Site and Experimental Design

**Figure 1:**
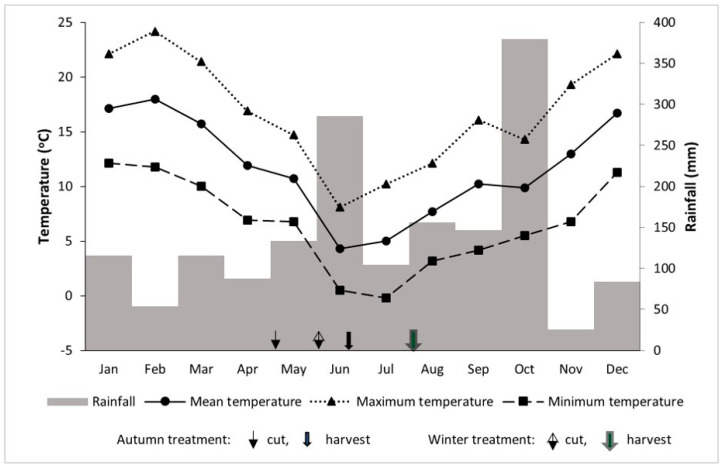
Monthly mean values for minimum and maximum temperatures and rainfall at Palmerston North, New Zealand in the year this work was undertaken. Arrows indicate cut and harvest dates.

The experimental protocol was approved by the Grasslands Animal Ethics Committee, AgResearch, Palmerston North, New Zealand (no. 11626). The experiment was conducted at the Grasslands Research Centre (40°22′51″ S, 175°36′51″ E), Palmerston North, New Zealand. Detailed climatic data for this region, including monthly minimum and maximum average temperatures, as well as rainfall, are presented in Figure 1.

A selection of nine ryegrass cultivars, representing the predominant choices in the region, were chosen for assessment. These cultivars, sourced from PGG Wrightson Seeds Limited and local seed distributors, are meticulously outlined in Table 1. The cultivars had been previously established in loam soil with a seeding rate of 20, 12, and 25 kg/ha for perennial, hybrid, and Italian ryegrass, respectively, and a plot size of 2 m × 3 m and maintained over a span of three years, undergoing mowing every four weeks during the summer and eight weeks during the winter, maintaining a consistent stubble height of 4 cm. No fertilizers were applied during this period. 

For the evaluation, Area 1 underwent the Autumn treatment, with mowing conducted on 3 May, followed by harvesting on 18 June after a regrowth period of 46 days. The Winter treatment (Area 2) involved mowing on 1 June, coinciding with the onset of winter, and harvesting on 2 August after 62 days of regrowth. In this experimental setup, each individual plot was regarded as the experimental unit. The design followed a 9 × 2 factorial arrangement within a randomised complete block design, encompassing the nine cultivars and the stockpiled conditions (autumn vs. winter).

Herbage samples (about 200 g DM each plot) were collected from the midpoint of the plot using an electric handpiece cutting at grazing level (about 4 cm above the ground). Out of each sample, a random selection of 30 fully expanded youngest leaves was procured for subsequent morphological and shear strength measurements. These leaves were stored between damp paper towels on ice until measured on the same day of sampling. The remaining portion of the sample was then stored at −16 °C, undergoing further mincing as described below for in situ incubation [15]. For DM determination, a subsample of 50 to 70 g DM from each cultivar was freeze-dried, after which it was ground for the estimation of NDF, acid detergent fibre (ADF), CP, soluble sugars and starch (SSS), lipid, ash, and metabolisable energy (ME) concentrations using near-infrared reflectance spectroscopy (NIRS). The spectra data spanning the range of 780 to 2500 nm were captured using an FT-NIR spectrophotometer (Bruker Optics, model MPA, Ettlingen, Germany). The calibration process was rooted in the spectra previously acquired from samples of known chemical composition, as documented by Corson et al. [22].

### 2.2. Leaf Morphology and Shear Strength

Ten leaves were selected at random to make a bundle, and three bundles were prepared for each plot. Within each bundle, leaf length was measured from the tip to the ligule (*n* = 3) [15] and the force required for shearing was determined for each bundle using a Warner–Bratzler meat tenderometer (*n* = 3) (General Electric Co., Canton, MA, USA) [23,24]. Three random leaves from each bundle were measured for width, thickness, and cross-sectional area (a total of nine leaves for each plot) using Vernier callipers, as described by Sun, Waghorn, and Clark [15].

### 2.3. In situ Incubations

To achieve particle sizes similar to ruminated forage [25], the frozen herbage was minced with the target particle size of 1–2 mm using a compact meat mincer as described by Sun, Waghorn, and Clark [15]. Briefly, prior to mincing, the forage was chopped to a length of 2–3 cm and kept in a freezer overnight. The mincer was also placed in a freezer to prevent the frozen herbage from thawing during mincing. Mincing was performed using a Kreft Compact meat mincer R70 (Kreft, GmbH, Schwelm, Germany). If thawing did happen during the mincing process, the mincer was placed in a freezer again for at least a half hour to cool down. Minced herbage was sealed in a plastic bag and stored at −16 °C.

Two mature fistulated nonlactating multiparous Friesian × Jersey cows with a body weight of 620 kg and a body condition score of 3 were employed for conducting in situ incubations. The cows were fed 10.4 kg DM of lucerne hay daily at 0800 and 1700 and had free access to water. The lucerne hay contained 172 g CP/kg DM and 531 g NDF/kg DM. The incubation methodology adhered to the protocol outlined by Sun, Waghorn, and Clark [15].

A total of 18 Dacron bags (10 cm × 10 cm with a pore size of 50 ± 15 μm; ANKOM technology, Macedon, NY, USA) were prepared per plot with each bag containing 5 g DM. Among these bags, 15 were placed into the ventral rumen in a lingerie bag and retrieved after 2, 4, 7, 9, 12, 24, and 72 h of incubation. Duplicate bags of each plot were retrieved at each incubation time and triplicate bags at 72 h. Bags designated for the identical incubation duration across the entire experimental set were concurrently introduced into the rumen and extracted simultaneously, following the established “all in, all out” methodology detailed by Sun and Waghorn [26]. Subsequent incubations of varying durations were executed on separate days. Immediately after incubation, all incubated bags were promptly immersed in an iced water bath. In addition, there were triplicate “0 h” bags, which were not incubated.

All Dacron bags were rinsed with tap water for approximately 2 min to cleanse their exteriors before being stored at a temperature of −16 °C. Before conducting the analysis, these bags were defrosted in 4 °C water, enclosed within lingerie bags, and subjected to a cold-water wash cycle using a commercial Simpson Genesis Automatic Washing Machine (Simpson Appliances, Penrose, New Zealand). This cycle lasted for 20 min on a ‘normal’ setting. Following this step, the polyester bags were separated from the lingerie bags and subjected to a 12-min wash using the ′delicates′ setting. The washed bags were determined for residual DM by oven drying at 65 °C for 48 h.

### 2.4. Degradation Kinetics

Dry matter degradation kinetics were calculated according to DM disappearance over time using the model described by Ørskov and McDonald [27] and modified by López et al. [28]:

*P* = *A* + *B* (1 − *e*^−*k* (*t*−*L*)^) (1)

where *P* is potential disappearance; *A* is the soluble fraction, being the proportion that can be washed out at 0 h; *B* is the insoluble but degradable fraction; *k* is the fractional disappearance rate of *B*; t is the incubation time (h), and *L* is the lag time (h). A nonlinear procedure was employed for analysis using SAS.

The effective degradability (*E*) was estimated using Equation (2) as follows:

*E* = *A* + *B* × *k*/(*k* + *k_p_*) (2)

where *k_p_* is the fractional passage rate, assumed to be 0.06/h when dairy cows were fed fresh ryegrass [29].

### 2.5. Statistical Analyses

The effects of harvest season and ryegrass cultivar or type on chemical composition, leaf morphology, leaf shear strength, and in situ degradation parameters were analysed using the two-way ANOVA with SAS [30] with the following statistical model:

Y_ij_ = µ + C_i_ +S_j_ + e_ij_ (3)

where Y_ij_ is the response, µ is the general mean, C_i_ is the fixed effect of cultivar or ryegrass type, S_j_ is the fixed effect of harvest season, and e_ij_ is the random error term.

In the context of chemical composition and degradation parameters, the experimental unit was defined as the plot, while for leaf morphology and shear strength, the individual leaf or a bundle of leaves was considered as the unit. Statistical significance was set at *p* ≤ 0.05.

## 3. Results

### 3.1. Chemical Composition

Both the ryegrass type and the season affected forage chemical composition. In Table 2, the average concentrations of NDF were 468 g/kg DM in Perennial, which was greater than that of Italian (414 g/kg DM; *p* < 0.05). The NDF concentration was greater in the Autumn treatment than in the Winter treatment (486 vs. 392 g/kg DM; *p* < 0.01). The ryegrass type had similar effects on ADF concentration as NDF. All Winter ryegrass cultivars were similar in CP concentration (208 g/kg DM) (*p >* 0.05), but Perennial had a lower CP concentration than Hybrid and Italian in Autumn (213, 255, and 270 g/kg DM, respectively; *p* < 0.05). Greater concentrations of soluble sugars and starch (SSS) were found in Italian than in Perennial and Hybrid (163 vs. 115 and 129 g/kg DM; *p* < 0.01), as well as in the Winter treatment than in the autumn treatment (156 vs. 116 g/kg DM). The estimated ME was also greater in Winter than in Autumn (12.6 vs. 11.9 MJ/kg DM; *p* < 0.05). Among ryegrass types, Italian had greater ME than Perennial and Hybrid (12.9 vs. 11.7 and 12.3 MJ/kg DM; *p* < 0.05). Different ryegrass types had similar lipid and ash concentrations (*p >* 0.05). 

### 3.2. Leaf Size, Cross-Sectional Area, Thickness, and Shear Strength

The leaves of Italian ryegrass were longer, wider, and thicker than those of the Perennial and Hybrid types, but the Perennial type had greater shear strength than the other two types. According to Table 3 and Table 4, the leaves of Italian were longer than Perennial and Hybrid, averaging 22.6, 14.2, and 15.4 cm, respectively (*p* < 0.01), and these differences were evident for both Autumn and Winter. Italian cultivars had wider (5.74 vs. 2.70 and 3.03 cm; *p* < 0.01) and thicker (0.301 vs. 0.233 and 0.240 cm; *p* < 0.05) leaves than Perennial and Hybrid. Consequently, the approximate cross-sectional area (assuming a rectangular form) was highest for Italian (1.82 mm^2^) compared to 0.64 mm^2^ for Perennial and 0.73 mm^2^ for Hybrid. Leaf width and thickness were similar between the two harvest dates. Perennial ryegrass had the greatest shear strength (0.83 kg/mm^2^), followed by Hybrid (0.61 kg/mm^2^) and Italian (0.31 kg/mm^2^) (*p* < 0.01). 

### 3.3. DM in situ Degradation

Soluble (A) and insoluble but degradable (B) fractions of DM and degradation kinetics are summarised in Table 5 and Table 6. The “A” fraction was greater in Italian (0.608) than in Perennial (0.443) and Hybrid (0.483) (*p* < 0.01). Consequently, Italian had a lower “B” fraction than Perennial and Hybrid (0.360 vs. 0.469 and 0.455; *p* < 0.01). There were no differences in degradation rates between Autumn and Winter (*p* = 0.15). Among ryegrass types, Italian tended to be degraded faster than Hybrid and Perennial (0.196 vs. 0.176 and 0.150/h; *p* = 0.06). 

## 4. Discussion

A dairy cow moves from late pregnancy to early lactation during the transition period, facing challenges in physiology and changes in nutritional requirements [5]. In the current study, we evaluated ryegrass cultivars and harvest season, providing useful information for cows pre- and post-calving in forage-based systems where fresh pasture fulfils the majority of nutritional requirements.

In pasture-based, seasonal dairy farming, cow calving predominantly takes place in late winter, synchronising their peak feed demand with the height of pasture growth, approximately six weeks following calving [31]. Conserved pastures, usually with supplements, are grazed during winter and at the commencement of lactation. The diet for pre-calving cows is often a mixture of conserved pastures, silage and/or brassicas, and other crops. For post-calving cows, the diet consists of conserved pastures and supplements [31,32]. After calving, the energy demand is doubled or tripled to sustain lactation and dietary CP requirements will be 18–20% of the DM [33]. An appropriate diet will facilitate the transition, and high intakes are essential to accommodate the increasing demand for energy and nutrients and to enable a resumption of cycling to achieve pregnancy within three months of calving [34].

The average CP concentration was 227 g/kg DM with no significant differences among the three ryegrass types, which exceeds cow requirements [8] and enables supplementation with low N feeds. However, the NDF concentration was higher in Perennial than in Hybrid and Italian, especially in the autumn growth, and was associated with greater shear strength, suggesting a lower quality and possibly lower intakes for cows fed Perennial than the other ryegrass types. The average concentration of soluble sugars and starch (SSS; 136 g/kg DM) was slightly lower in the autumn vs. winter regrowth, but the calculated non-structural carbohydrates (NSCs; total DM less the NDF, CP, lipid, and ash) differed substantially between seasons. The calculated NSCs in autumn were much less than that for the winter treatment, suggesting that the nutritional composition of ryegrass can vary significantly depending on the season. As a result, these seasonal variations should be considered when planning livestock feeding strategies to ensure optimal nutrition for their cows.

Our analyses demonstrate differences between cultivars, especially ryegrass types, with clear evidence of the superior nutritive characteristics of Italian and Hybrid relative to Perennial, supported by their predicted ME values. However, greater differences in composition and other characteristics were evident when comparing the grasses grown in late autumn and in winter. The lower concentration of NDF and the higher concentrations of NSCs in winter regrowth are likely to have significant implications for feeding values (intake × feed quality), especially for Perennial ryegrasses. The intake of forages can be limited by rumen fill, and this is affected by the amount of fibre and the rate of particle breakdown, affecting the outflow to the lower digestive tract [3,33]. Furthermore, the poor efficiency of autumn vs. spring pasture for production [35] has been attributed to low concentrations of NSCs (contributing to a 24% lower volatile fatty acid yield) and an excess CP, which has a low energy yield relative to carbohydrates, and incurs a metabolic cost for disposal as urea [3,36]. The Italian cultivars tended to have the lowest NDF and shear strength and the least change between autumn and winter treatments, suggesting a high feeding value for dairy cows and other ruminants.

Nutritional requirements differ greatly for pre- and post-calving cows. Before calving, a high-fibre diet, such as Perennial ryegrasses or straw [37], is recommended to stimulate chewing and to increase intake for post-calving by developing rumen musculature [38]. Conversely, the post-calving diet should be easily and rapidly degraded and contain about 35% NDF [3] to avoid acidosis with increasing DM intake. The pasture’s CP concentration should be high enough to achieve an adequate total diet CP concentration when energy supplements with a lower CP concentration are offered [4,39]. The results presented here suggest that Perennial ryegrass is suitable for pre-calving cows, while Italian and Hybrid are suitable for post-calving cows.

The suggestion that the Perennial be fed to pre-calving cows and the Italian or Hybrid to post-calving is also supported by their shear strength and degradation characteristics. Shear strength was associated with their resistance to particle size reduction by animal chewing. Shear strength is an indicator to show how easily leaves are broken down during chewing and rumination [23,40] and is associated with DM intake and digestibility [41]. Previously, the evaluation of shear strength among ryegrass cultivars [42] and other forages [43] was reported. Although cultivars varied the proportion of DM in the “A” fraction of winter growth, values were higher for all of the Italian cultivars (average 0.572) than for other cultivars (Table 5), and Italian cultivars had the greatest average degradation rate (0.171/h), confirming that it is the best among the three ryegrass types for a high DM intake in early lactation cows.

In addition to climate variables, the changes in nutritional requirements associated with physiology are another key factor for dairy farmers when managing pastures. In most cases, supplements are widely used to fill the shortages of pasture. Forage species, cultivars, and supplements available to New Zealand dairy farmers have increased with increasing profitability. Better use of pastures and supplements relies on a better understanding of their feeding value and cow requirements. The characteristics of dietary components, both chemical and physical, could affect animal nutrition and eventually production, especially during the transition period when the cow’s physiological state changes rapidly. The results presented here confirm significant differences among ryegrass types and cultivars, suggesting a potential strategy to optimise pasture management and feeding strategy over winter and early spring in New Zealand.

It is of paramount importance to recognise that the scope of this investigation encompassed a mere one-year study duration. Such brevity underscores the substantial year-to-year fluctuations inherent in climatic variables, which invariably impact the findings. Consequently, the scope for interpreting the results of this study is inherently constrained. Moreover, it is imperative to underscore the imperative for further inquiry to comprehensively ascertain the potential impacts of pasture management practices on ryegrass responses. Notably, the current study hinged upon mechanical harvesting as the method for forage removal, thereby potentially engendering dissimilar regrowth patterns compared to animal grazing scenarios. It is thus prudent to exercise caution in extrapolating the findings of this study to the broader context of grazing dairy animals in real farm conditions, particularly considering the nuanced diversities across determinants, such as parity.

## 5. Conclusions

The chemical composition and in situ degradation kinetics of ryegrass grown in late autumn or early winter in a temperate climate differed among types and cultivars. Perennial ryegrass exhibits a higher fibre content in comparison to the other two ryegrass types, whereas Italian ryegrass displays a greater proportion of soluble dry matter and a faster degradation rate of insoluble yet degradable dry matter when compared to hybrid and perennial ryegrasses. Additionally, Autumn ryegrass showcases elevated levels of crude protein and fibre but a reduced concentration of soluble carbohydrates in contrast to Winter ryegrass. Understanding these differences will assist with planning and grazing management before and after calving in pasture-based seasonal dairy farming and could be used to optimise dairy cow nutrition.

## Figures and Tables

**Table 1:** Ryegrass types and cultivars that were evaluated and their general characteristics.

Cultivar	Type	Scientific Name	Ploidy	Characteristics
Samson	Perennial	*Lolium perenne* L.	2	High yielding, good summer growth and quality, low aftermath heading
Impact		*L. perenne* L.	2	Late flowering, out-of-season growth, creeping stem
Coronet		*L. perenne* L.	2	turf ryegrass
Supreme	Hybrid	*L. perenne × Lolium multiflorum*	2	Long rotation, fine–medium leaves
Manawa		*L. perenne × L. multiflorum*	2	Erect growth, large wide-leafed tillers, persists for 6–8 years, good winter production
Greenstone		*L. perenne × (L. perenne × L. multiflorum)*	4	Vigorously tillered, large tillers, and erect growth
Warrior	Italian	*L. multiflorum* L.	2	Extended spring/summer production, high winter growth, densely tillered, fine-leaved
Tama		*L. multiflorum* L.	4	Very high winter and early production
Moata		*L. multiflorum* L.	4	Persists up to 2 years

**Table 2:** Chemical composition of ryegrasses (g/kg dry matter (DM), unless indicated) harvested after growth in late autumn and winter, estimated using near-infrared reflectance spectroscopy.

Harvest Season	Type	Cultivar	NDF ^†^	ADF ^†^	CP ^†^	SSS ^†^	Lipid	Ash	ME ^†^ (MJ/kg DM)
Autumn ^‡^	Perennial	Samson	507	259	211	124	34	79	11.9
		Impact	521	274	223	94	29	75	11.2
		Coronet	576	302	204	86	32	65	9.9
		Mean	535 ^a^ *	278 ^a^	213 ^c^	101 ^b^	32 ^b^	73 ^c^	11.0 ^c^
	Hybrid	Supreme	492	246	251	117	34	88	12.1
		Manawa	484	285	261	100	31	69	11.3
		Greenstone	468	232	253	128	38	89	12.6
		Mean	481 ^b^	254 ^b^	255 ^a^	115 ^ab^	34 ^b^	82 ^b^	12.0 ^b^
	Italian	Warrior	490	259	232	127	34	80	11.8
		Tama	423	222	290	108	38	90	13.0
		Moata	415	236	287	160	39	105	13.3
		Mean	443 ^c^	239 ^b^	270 ^a^	132 ^a^	37 ^a^	92 ^a^	12.7 ^a^
		SEM ^†^ (Type)	19.1	13.4	11.3	12.0	1.4	6.4	0.49
Winter ^‡^	Perennial	Samson	416	227	202	140	37	96	12.5
		Impact	399	210	233	125	42	105	12.6
		Coronet	390	230	239	122	43	107	12.0
		Mean	402 ^a^	222	225 ^a^	129 ^b^	41 ^c^	103 ^a^	12.4 ^b^
	Hybrid	Supreme	401	223	218	131	40	99	12.4
		Manawa	389	216	228	150	44	99	12.4
		Greenstone	381	213	213	147	41	97	12.8
		Mean	390 ^b^	217	220 ^a^	143 ^b^	42 ^a^	98 ^b^	12.5 ^b^
	Italian	Warrior	399	229	192	159	39	94	12.6
		Tama	375	212	180	202	37	84	13.3
		Moata	383	219	170	224	40	85	13.2
		Mean	386 ^b^	220	181 ^b^	195 ^a^	39 ^b^	88 ^c^	13.0 ^a^
		SEM (Type)	7.1	4.9	7.8	12.0	1.4	2.8	0.21

^†^ NDF = Neutral detergent fibre; ADF = Acid detergent fibre; CP = Crude protein; SSS = Soluble sugars and starch; ME = Metabolisable energy (MJ/kg DM); SEM = Standard error of the mean. * Different letters on the shoulder of values within a column for each harvest season indicate a significant difference among ryegrass types (*p* < 0.05). ^‡^ Autumn treatment: cut on 3 May and harvested on 18 June; Winter treatment: cut on 1 June and harvested on 2 August.

**Table 3:** Leaf morphology and shear strength of ryegrasses after autumn growth.

Type	Cultivar	Leaf Length (cm)	Leaf Width (mm)	Leaf Thickness (mm)	Cross-Sectional Area (mm^2^)	Shear Force (kg/10 Leaves)	Shear Strength (kg/mm^2^)
Number of analyses per cultivar		3	9	9	9	3	1
Perennial	Samson	13.3	2.41	0.222	0.535	5.7	1.07
	Impact	19.3	2.43	0.217	0.526	5.4	1.03
	Coronet	10.7	1.9	0.205	0.389	3.3	0.85
	Mean	14.4 ^c^ *	2.25 ^c^	0.215 ^c^	0.483 ^b^	4.8 ^a^	0.98 ^a^
Hybrid	Supreme	20.7	2.82	0.246	0.693	4.7	0.68
	Manawa	12.7	2.55	0.208	0.529	3.1	0.59
	Greenstone	19.3	2.9	0.264	0.766	4.8	0.63
	Mean	17.6 ^b^	2.76 ^b^	0.239 ^b^	0.663 ^b^	4.2 ^b^	0.63 ^b^
Italian	Warrior	18.7	3.1	0.217	0.671	3.8	0.57
	Tama	30.3	8.01	0.354	2.836	4.3	0.15
	Moata	23.3	5.84	0.307	1.794	3.5	0.2
	Mean	24.1 ^a^	5.65 ^a^	0.293 ^a^	1.767 ^a^	3.9 ^b^	0.31 ^c^
	SEM ^†^ (Type)	1.41	0.297	0.0113	0.1379	0.35	0.085
	SEM (Cultivar)	2.83	0.827	0.0255	0.3642	0.57	

^†^ SEM = Standard error of the mean. * Different letters on the shoulder of values within a column indicate a significant difference among ryegrass types (*p* < 0.05).

**Table 4:** Leaf morphology and shear strength of evaluated ryegrasses after winter growth.

Type	Cultivar	Leaf Length (cm)	Leaf Width (mm)	Leaf Thickness (mm)	Cross-Sectional Area (mm^2^)	Shear Force (kg/10 leaves)	Shear Strength (kg/mm^2^)
Number of analyses per cultivar		3	9	9	9	3	1
Perennial	Samson	15.0	3.55	0.278	0.987	5.3	0.54
	Impact	15.5	3.15	0.239	0.752	5.6	0.74
	Coronet	11.2	2.80	0.237	0.662	4.9	0.74
	Mean	13.9 ^b^ *	3.17 ^b^	0.251 ^b^	0.800 ^b^	5.3 ^a^	0.67 ^a^
Hybrid	Supreme	13.0	3.41	0.246	0.841	5.2	0.62
	Manawa	13.1	3.30	0.217	0.714	4.5	0.63
	Greenstone	13.7	3.23	0.260	0.840	4.3	0.51
	Mean	13.3 ^b^	3.31 ^b^	0.241 ^b^	0.798 ^b^	4.7 ^b^	0.59 ^a^
Italian	Warrior	16.3	3.60	0.246	0.886	4.0	0.45
	Tama	25.7	7.65	0.341	2.608	5.5	0.21
	Moata	21.5	6.26	0.340	2.125	5.5	0.26
	Mean	21.2 ^a^	5.84 ^a^	0.309 ^a^	1.873 ^a^	5.0 ^ab^	0.31 ^b^
	SEM ^†^ (Type)	0.49	0.219	0.0120	0.0035	0.21	0.061
	SEM (Cultivar)	1.77	0.700	0.0212	0.3026	0.35	

^†^ SEM = Standard error of the mean. * Different letters on the shoulder of values within a column indicate a significant difference among ryegrass types (*p* < 0.05).

**Table 5:** Dry matter fractions and degradation parameters of ryegrasses after autumn growth.

Type	Cultivar	Soluble Fraction (%)	Insoluble Degradable Fraction (%)	Fractional Disappearance Rate (/h)	Lag Time (h)	Indigestible Fraction (%)	Effective Degradability (%)
Perennial	Samson	48.1	42.6	0.207	4.8	9.2	81.2
	Impact	45.9	44.8	0.134	3.4	9.2	76.9
	Coronet	28.1	48.9	0.095	3.3	23.0	58.0
	Mean	40.7 ^b^ *	45.4 ^a^	0.145 ^c^	3.8 ^b^	13.8 ^a^	72.0 ^c^
Hybrid	Supreme	53.1	40.7	0.186	4.7	6.2	83.9
	Manawa	35.2	49.5	0.180	5.1	15.2	72.4
	Greenstone	51.9	43.0	0.200	4.7	5.1	85.0
	Mean	46.7 ^a^	44.4 ^a^	0.189 ^b^	4.8 ^a^	8.8 ^b^	80.4 ^b^
Italian	Warrior	55.6	38.3	0.198	5.0	6.1	85.0
	Tama	68.4	29.0	0.214	3.7	2.6	91.1
	Moata	68.6	29.8	0.250	3.2	1.6	92.6
	Mean	64.2 ^a^	32.4 ^b^	0.221 ^a^	4.0 ^b^	3.4 ^c^	89.6 ^a^
	SEM ^†^ (Type)	5.52	2.55	0.0212	0.42	3.32	4.95

^†^ SEM = Standard error of the mean. * Different letters on the shoulder of values within a column indicate a significant difference among ryegrass types (*p* < 0.05).

**Table 6:** Dry matter fractions and degradation parameters of ryegrasses after winter growth.

Type	Cultivar	Soluble fraction (%)	Insoluble Degradable Fraction (%)	Fractional Disappearance rate (/h)	Lag Time (h)	Indigestible Fraction (%)	Effective Degradability (%)
Perennial	Samson	45.9	50.7	0.152	2.9	3.4	82.2
	Impact	49.0	48.0	0.157	2.8	2.9	83.8
	Coronet	48.7	46.1	0.157	3.0	5.2	82.0
	Mean	47.9 ^b^ *	48.3 ^a^	0.155 ^b^	2.9	3.8 ^a^	82.7 ^b^
Hybrid	Supreme	49.2	47.5	0.166	3.1	3.3	84.1
	Manawa	48.2	48.1	0.150	2.9	3.6	82.6
	Greenstone	51.9	44.5	0.170	2.8	3.6	84.8
	Mean	49.8 ^b^	46.7 ^a^	0.162 ^ab^	2.9	3.5 ^ab^	83.8 ^b^
Italian	Warrior	53.0	43.1	0.166	3.3	3.9	84.6
	Tama	60.0	38.0	0.144	2.6	2.1	86.8
	Moata	59.2	37.9	0.204	2.7	2.9	88.5
	Mean	57.4 ^a^	39.7 ^b^	0.171 ^a^	2.9	3.0 ^b^	86.6 ^a^
	SEM ^†^ (Type)	1.56	1.41	0.0106	0.14	0.49	0.85

^†^ SEM = Standard error of the mean. * Different letters on the shoulder of values within a column indicate a significant difference among forage types (*p* < 0.05).

## Data Availability

The datasets used and/or analysed during the current study are available from the first author upon reasonable request.
